# Characteristics, Relationships, and Anatomical Basis of Leaf Hydraulic Traits and Economic Traits in Temperate Desert Shrub Species

**DOI:** 10.3390/life14070834

**Published:** 2024-06-29

**Authors:** Fengsen Tan, Wenxu Cao, Xu Li, Qinghe Li

**Affiliations:** State Key Laboratory of Efficient Production of Forest Resources, Key Laboratory of Tree Breeding and Cultivation of National Forestry and Grassland Administration, Research Institute of Forestry, Chinese Academy of Forestry, Beijing 100091, China; tanfengsen@126.com (F.T.); lyscwx@caf.ac.cn (W.C.); lxu@caf.ac.cn (X.L.)

**Keywords:** hydraulic efficiency, hydraulic safety, nutritional element content, leaf thickness, specific leaf area, anatomical structure

## Abstract

Shrubs are a key component of desert ecosystems, playing a crucial role in controlling desertification and promoting revegetation, yet their growth is often impeded by drought. Leaf hydraulic traits and economic traits are both involved in the process of water exchange for carbon dioxide. Exploring the characteristics, relationships, and anatomical basis of these two suites of traits is crucial to understanding the mechanism of desert shrubs adapting to the desert arid environment. However, the relationship between these two sets of traits currently remains ambiguous. This study explored the leaf hydraulic, economic, and anatomical traits of 19 desert shrub species. The key findings include the following: Relatively larger LT values and smaller SLA values were observed in desert shrubs, aligning with the “slow strategy” in the leaf economics spectrum. The relatively high P50_leaf_, low HSM_leaf_, negative TLP_leaf_, and positive HSM_tlp_ values indicated that severe embolism occurs in the leaves during the dry season, while most species were able to maintain normal leaf expansion. This implies a “tolerance” leaf hydraulic strategy in response to arid stress. No significant relationship was observed between P50_leaf_ and K_max_, indicating the absence of a trade-off between hydraulic efficiency and embolism resistance. Certain coupling relationships were observed between leaf hydraulic traits and economic traits, both of which were closely tied to anatomical structures. Out of all of the leaf traits, LT was the central trait of the leaf traits network. The positive correlation between C content and WP_leaf_ and HSM_leaf_, as well as the positive correlation between N content and HSM_tlp_, suggested that the cost of leaf construction was synergistic with hydraulic safety. The negative correlation between SLA, P content, GCL, and SAI suggested a functional synergistic relationship between water use efficiency and gas exchange rate. In summary, this research revealed that the coupling relationship between leaf hydraulic traits and economic traits was one of the important physiological and ecological mechanisms of desert shrubs for adapting to desert habitats.

## 1. Introduction

Shrubs are a key component of the desert ecosystem and play an important role in maintaining the functions and structure of the desert ecosystem [1,2]. Desert shrubs refer to a type of low-growing, drought-resistant, woody plant that grows in desert environments and is characterized by the absence of a distinct single trunk, and these are the main dominant plants in arid desert systems [3,4]. Elucidating the ecological adaptation strategies of desert shrubs is of great significance for predicting the dynamic changes of vegetation in desert ecosystems under future climate change, and it can also provide a theoretical basis for the protection of vegetation and the ecological restoration of degraded desert ecosystems. 

Leaves are the main organs for photosynthesis and transpiration in plants. Leaf functional traits are one of the most important groups of plant functional traits, and they largely reflect a plant’s strategies for adaptation to its environment [5,6,7] Leaf hydraulic and economic traits have received the greatest attention among the leaf functional traits. Leaf hydraulic traits, including both hydraulic efficiency and hydraulic safety, are key characteristics reflecting water adaptation mechanisms in terrestrial plants [8,9,10,11]. Leaf economic traits include leaf thickness, leaf mass per area (or specific leaf area), nitrogen and phosphorus content, maximum photosynthetic capacity, and leaf lifespan, among others, and form the so-called worldwide leaf economic spectrum, which is thought to influence plant growth and survival [12,13,14]. 

The direction of leaf evolution is not to optimize individual key traits but to optimize the combination of different traits to better adapt to historical and current environmental changes [15]. Although leaf economic traits and hydraulic traits both involve the process of exchanging water for carbon dioxide, there is still debate about whether these two sets of traits are interdependent or independent [16]. Some studies suggest that the isohydric–anisohydric continuum of plants is interconnected with the leaf economic spectrum, and this connection is closely related to leaf structure [17,18]. Anisohydric species correspond to “fast strategy” species, which need to maintain high stomatal conductance to sustain efficient rates of gas exchange and photosynthesis [19]. High stomatal conductance implies a high transpiration rate, which relies on efficient water conduction rates [20,21]. High hydraulic conduction efficiency is associated with high stomatal density, stomatal conductance, vein density, and large xylem vessel diameter in the leaf veins, which in turn increases the risk of gas embolism in the leaves [18,22]. These plants tend to have lower leaf construction costs, lower specific leaf weights, and shorter leaf lifespans. In contrast, isohydric species correspond to “slow strategy” species, which tend to close their stomata earlier in response to dry conditions, resulting in a more moderate decrease in water potential. These species display characteristics of conservative resource use and high hydraulic safety [23]. There are also studies that suggest economic traits and hydraulic traits have evolved relatively independently, allowing for a greater variety of plant trait combinations [24,25]. Compared to a single trait dimension, multiple trait dimensions facilitate plants to adapt to multiple niche dimensions, promote a diversity of plant strategies, and are beneficial for species coexistence [24]. Yin et al. (2018) suggested that the correlation between hydraulic traits and economic traits may be related to the availability of water in the environment. In areas with high species richness and complex environments, hydraulic traits and economic traits are relatively independent, while in arid habitats, economic traits and hydraulic traits tend to have a stable coupling relationship [26].

Structure determines function. Both hydraulic and economic traits of plants are closely related to leaf structure [15,27,28,29]. The complexity of leaf structure, including cross-sectional, stomatal, and venation patterns, affects the internal gas exchange and photosynthetic processes within the leaf [30,31,32,33]. Therefore, revealing the correlation between leaf hydraulic traits and economic traits with the anatomical structure helps science to understand the physiological mechanisms of leaf adaptation to the environment. 

Current research on the relationship between hydraulic traits and economic traits is mainly focused on woody plants with larger leaves, utilizing the structural traits of veins and stomata as hydraulic indicators [27,34]. However, there is a lack of in-depth studies regarding the relationship between economic traits and important hydraulic physiological traits, such as K_max_ (maximum conductance in leaves), P50_leaf_ (the water potential inducing 50% loss of maximum conductance in leaves), and TLP_leaf_ (leaf water potential at turgor loss point). The diverse morphology of desert shrub leaves, including fleshy, leathery, and needle-like types with smaller leaf areas, complicates the understanding of their hydraulic and economic traits. Additionally, leaf hydraulic traits and economic traits may also be influenced by the hydraulic traits of other organs [35,36,37,38]. The morphology of desert shrub stems and roots differs significantly from other forest ecosystems [39], which further adds complexity to the relationship between leaf hydraulic traits and economic traits. Therefore, this study investigated the hydraulic traits, economic traits, and anatomical structure of 19 typical desert shrubs (Table 1). We endeavored to address the following questions: (1) What are the typical characteristics of leaf hydraulic traits and economic traits in desert shrubs, and (2) what is the relationship between hydraulic traits and economic traits and their anatomical basis in desert shrubs?

## 2. Materials and Methods

### 2.1. Study Sites and Species

**Figure 1:**
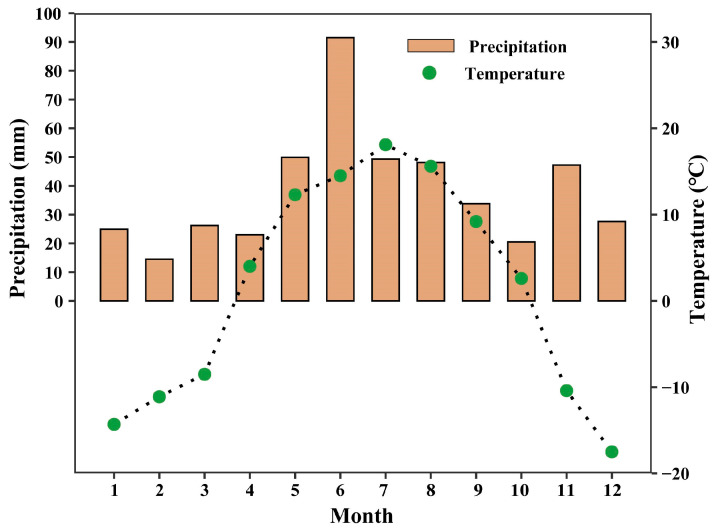
Monthly precipitation (bars) and average atmospheric temperature (circles) in the study area in 2022.

This research was conducted in a semi-arid desert area of Inner Mongolia, China (39°20′ N~40°29′ N; 106°39′ E~107°28′ E) in August and September 2022. Monthly precipitation and average atmospheric temperature in the study area in 2022 are shown in Figure 1. The region is marked by a typical temperate continental desert climate, featuring brief, hot summers and extended, cold winters. Across the sites, the mean annual precipitation (MAP) ranges from 140 to 250 mm, and the mean annual evaporation hovers around 2800 mm, with most rainfall concentrated in the summer months of July to September. The mean annual air temperature (MTP) is 7.8 °C. The predominant soil is sandy in this area. In general, the study area is characterized by the prevalence of strong winds and sand, dryness, strong solar radiation, and extreme fluctuations in temperature [40]. The vegetation is dominated by semi-shrubs, shrubs, and grasses. Shrubs in this region display a pronounced seasonal growth cycle, with a decrease in metabolic processes during the winter months and a period of vigorous growth during spring and summer. In total, 19 typical desert shrub species belonging to 8 families and 14 genera were selected in this study (Table 1 and Table 2). The species selected for this study were perennial with high relative abundance and could be used to measure traits in this study. The species selected in this study included most of the typical desert shrubs in the research area. 

### 2.2. Method

#### 2.2.1. Determination of Anatomical Traits

During the growing season, samples were collected from 5 individuals of each species. Ten healthy sunlit leaves were collected from each individual and immediately placed in an FAA fixative for preservation. Due to the leaves of *Caragana brachypoda* being too small and hairy, the anatomical structure traits of *C. brachypoda* were not accurately determined in this study, and thus, data for this species were not included in the analysis of anatomical structures.

For each species, 2 healthy mature leaves were selected from each of the 5 individuals collected. Hand-sectioning was used to prepare transverse sections of the leaves, which were then observed and photographed under a light microscope (Leica DM2500 Leica Microsystems, Wetzlar, Germany). Three fields of view were captured for each leaf (a total of 30 images per species). The leaf cuticle thickness (TC, μm), spongy tissue thickness (Sp, μm), epidermis tissue thickness (Tep, μm), and palisade tissue thickness (Tpa, μm) were measured using ImageJ software (Fiji v1.52n). The ratio of spongy tissue thickness to palisade tissue thickness (Sp/Tpa) was calculated as the ratio of Sp to Tpa. The ratio of cuticle thickness to leaf thickness (TCf, %) was calculated as the ratio of TC to the sum of the thicknesses of Tpa, Sp, Tep, and TC.

For each species, 2 healthy mature leaves were selected from each of the 5 individuals collected. The leaves were immersed in bleach until the cuticle and mesophyll could be easily separated [43]. After washing the leaves with water, they were mounted on microscope slides. These sections were then observed and photographed under a light microscope (Leica DM2500, Germany). Three fields of view were captured for each leaf (a total of 30 images per species). The leaf vein density (LVD) and midrib diameter (MD) were measured using ImageJ software.

Stomatal characteristics were determined using the nail polish impression method [44]. For each species, 2 healthy mature leaves were selected from each of the 5 individuals collected. Since all species in this study have stomata on both sides of the leaves, the sunny and shady sides of the leaves were measured separately. After the leaf hairs were removed, clear nail polish was applied evenly. Once the nail polish had dried, transparent tape was stuck onto the surface of the nail polish and then carefully peeled off. Transparent tape with stomatal features was attached to slides, observed, and photographed under a light microscope (Leica DM2500, Germany). Three fields of view were captured for one side of each leaf (30 pictures each of the shaded side and the sunny side of each species). The sum of stomatal density (SD) and average guard cell length (GCL) were determined using ImageJ software. The stomatal area index (SAI) was calculated according to the following formula: SAI = SD × GCL^2^. Potential maximum stomatal conductance to water vapor (g_max_, mol m^−2^ s^−1^) was calculated according to eqn 2 in Franks et al. (2009) [44]. Due to the small size of the *Potaninia mongolica* leaves and the difficulty in removing the hairs, the stomatal characteristics of *Potaninia mongolica* were not determined.

#### 2.2.2. Determination of Economic Traits

For each species, 30 to 50 (depending on the leaf size) healthy mature leaves were selected from each of 5 healthy, sun-exposed branches from different individuals. After flattening the leaves, they were scanned using a flatbed scanner (Epson V850Pro, Jawa Barat, Indonesia), and the leaf area of each replicate was measured using ImageJ software. After scanning, the leaves were dried in an oven at 70 °C for 48 h to determine their dry weight. Specific leaf area (SLA, m^2^ Kg^−1^) was calculated as the area of leaves per unit mass. The dried leaves were then ground and used to measure the leaf carbon, nitrogen, and phosphorus content. Leaf nitrogen content (N, mg g^−1^) and leaf carbon content (C, mg g^−1^) were determined using an elemental analyzer (Eurovector EA3100 EuroVector, Pavia, Italy), and leaf phosphorus content (P, mg g^−1^) was measured by microwave digestion-ICP-MS method. The leaf nitrogen to phosphorus ratio (N:P) was calculated as the ratio of N content to P content. The leaf thickness (LT, μm) was calculated as the sum of the thicknesses of Tpa, Sp, Tep, and TC.

#### 2.2.3. Determination of Hydraulic Traits

Leaf hydraulic traits data were sourced from the unpublished article of Tan et al. (2024) [45]. The measurement methods for hydraulic traits are in the Supplementary Materials.

### 2.3. Data Analysis

Principal component analysis (PCA) and network analysis were used to determine the multivariate associations of leaf traits in R 4.1.3 software. The relationships between leaf hydraulic traits, economic traits, and anatomical traits were examined by analyzing the first principal component scores from each set of traits [24]. Pearson correlation analysis for pairwise leaf traits and the first principal component scores of the three sets of leaf traits was performed using SPSS 20.0 software. Principal component plots were plotted in R 4.1.3 software, network plots in Gephi 0.10 software, and correlation plots in SigmaPlot 12.5 software.

## 3. Results

### 3.1. Characteristics of Leaf Hydraulic Traits

The mean TLP_leaf_ was −3.81 MPa, ranging from −6.21 to −1.98 MPa. The mean HSM_tlp_ was 0.65 MPa, ranging from −178 to 3.05 MPa. The mean P50_leaf_ was −0.98 MPa, ranging from −0.73 to −1.23 MPa. HSM_leaf_ was negative for all species, averaging −2.00 MPa, with a range from −3.59 to −0.74 MPa. The mean K_max_ was 451.86 mmol Kg^−1^ MPa^−1^ s^−1^, with a range from 157.31 to 716.84 mmol Kg^−1^ MPa^−1^ s^−1^ (Table 3).

Among leaf hydraulic traits, the correlation between K_max_ and P50_leaf_ was not significant. K_max_ was significantly positively correlated with Cm but not with Cs. HSM_leaf_ was significantly positively correlated with WP_leaf_, significantly negatively correlated with Cs, and was not correlated with P50_leaf_. HSM_tlp_ was extremely significantly negatively correlated with TLP_leaf_ and was not correlated with WP_leaf_ (Table S1).

### 3.2. Characteristics of Leaf Economic Traits

The mean values of SLA, C content, N content, P content, N:P, and LT were 13.09 m^2^ Kg^−1^, 28.47 mg g^−1^, 434.55 mg g^−1^, 1.56 mg g^−1^, 19.39, and 482.35 μm, respectively. The ranges of variations were from 7.10 to 29.14 m^2^ Kg^−1^, 18.305 to 42.39 mg g^−1^, 295.74 to 523.66 mg g^−1^, 0.87 to 42.39 mg g^−1^, 10.36 to 27.71, and 212.66 to 1382.63 μm, respectively (Table 2).

Among leaf hydraulic traits, SLA was significantly positively correlated with P content and negatively correlated with N:P. LT was significantly and negatively correlated with C content. There was also a significant positive correlation between N content and P content (Table S2).

### 3.3. Relationships between Leaf Hydraulic Traits, Economic Traits, and Anatomical Traits

**Figure 2:**
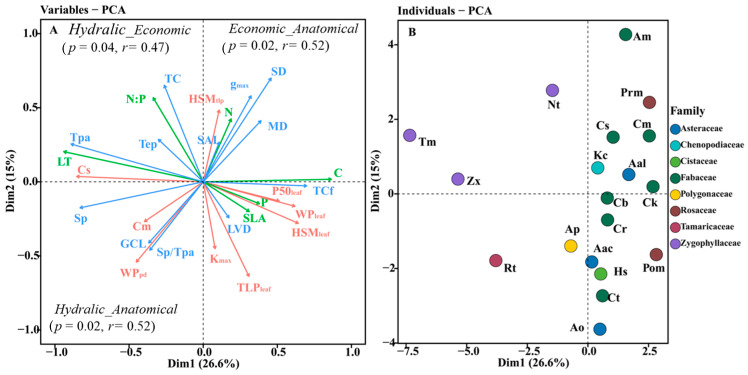
Principal component analysis for (**A**) leaf traits and (**B**) desert shrub species for the first two axes. The trait loading plot shows leaf hydraulic traits as red lines, leaf economics traits as green lines, and leaf anatomical traits as blue lines. The PCA ordination relationships between hydraulic traits, economic traits, and anatomical traits were represented by the correlations between the first principal component scores of each set of traits. Trait abbreviations follow Table 3.

Principal component analysis showed that the first axis explained 26.6% of the total variance, with C content, TCf, HSM_leaf_, WP_leaf_, and P50_leaf_ showing large positive loadings, mainly related to structural drought tolerance, and LT, Tpa, Cs, and Sp showing large negative loadings, mainly related to water storage capacity. The second principal component explained 15.0% of the total variance, with TC, N:P, SD, and HSM_tlp_ having large positive loadings and TLP_leaf_, WP_pd_, GCL, and Sp/Tpa having large negative loadings (Figure 2).

**Figure 3:**
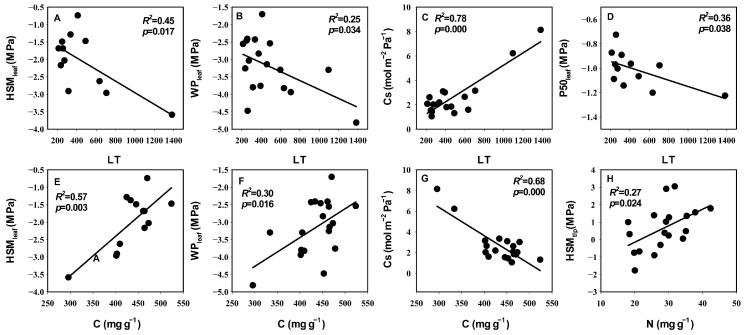
Relationships between hydraulic traits and economic traits. (**A**–**D**) Correlation of LT with HSM_leaf_, WPl_eaf_, Cs, and P50_leaf_. (**E**–**H**) Correlation of N with HSM_leaf_, WPl_eaf_, Cs, and HSM_leaf_. The coefficients of determination (*R*^2^) and significance levels (*p*) of linear regression are shown. Trait abbreviations follow Table 3.

**Figure 4:**
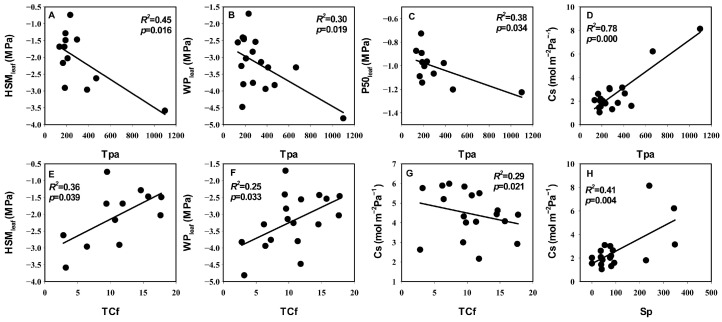
Relationships between hydraulic traits and anatomical traits. (**A**–**D**) Correlation of Tpa with HSM_leaf_, WPl_eaf_, P50_leaf_, and Cs. (**E**–**G**) Correlation of TCf with HSM_leaf_, WPl_eaf_, Cs. (**H**) Correlation of Cs with HSM_leaf_. The coefficients of determination (*R*^2^) and significance levels (*p*) of linear regression are shown. Trait abbreviations follow Table 3.

**Figure 5:**
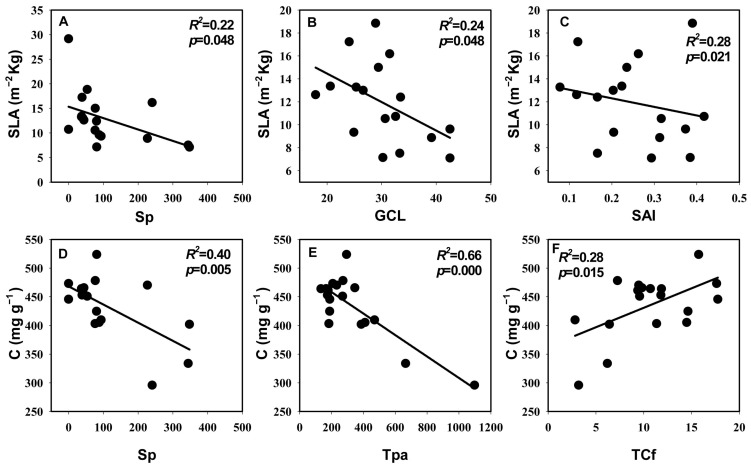
Relationships between economic traits and anatomical traits. (**A**–**C**) Correlation of SLA with Sp, GCL, and SAI. (**D**–**F**) Correlation of C with Sp, Tpa, TCf. The coefficients of determination (*R*^2^) and significance levels (*p*) of linear regression are shown. Trait abbreviations follow Table 3.

In the correlation analysis, the significant correlations (*p* = 0.04, *r* = 0.47) between the first principal component scores of leaf economic traits and hydraulic traits indicated the coupling relationship between those two groups’ traits. The first principal component scores of both leaf hydraulic traits and economic traits were also significantly positively correlated with the first principal component scores of anatomical traits (*p* values were both 0.02; *r* values were both 0.52) (Figure 2A). Specifically, C content was extremely significantly negatively correlated with Cs and significantly positively correlated with WP_leaf_ and HSM_leaf_. N content was significantly positively correlated with HSM_tlp_. LT was extremely significantly positively correlated with both Cs and significantly negatively correlated with P50_leaf_, HSM_leaf_, and WP_leaf_. SLA was significantly negatively correlated with Sp, GCL, and SAI. C content was extremely significantly negatively correlated with LT, Tpa, and Sp, and significantly positively correlated with TCf. P50_leaf_, WP_leaf_, and HSM_leaf_ were significantly negatively correlated with Tpa and LT, respectively. WP_leaf_ and HSM_leaf_ were significantly positively correlated with TCf. MD was significantly negatively correlated with Cm. Cs was extremely significantly positively correlated with Sp and significantly negatively correlated with TCf. Cs was extremely significantly positively correlated with both Tpa and LT (Figure 3, Figure 4 and Figure 5).

**Figure 6:**
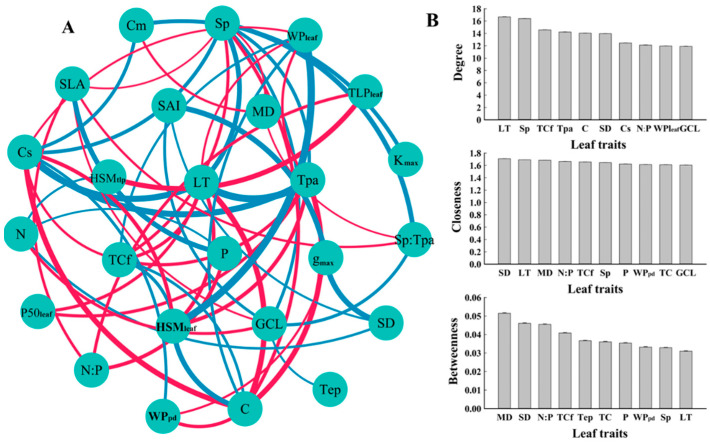
Correlation network for leaf traits of desert shrubs. (**A**) Leaf trait network. Blue and red lines represent positive and negative correlations, respectively. The thickness of the line represents the strength of the correlation. (**B**) Network node characteristics, degree, tightness, and median of the top 10 ranked traits. Trait abbreviations follow Table 3.

The correlation network analysis revealed that LT, Sp, and TCf exhibited the highest degrees; SD, LT, and MD demonstrated the highest closeness; MD, SD, and N:P exhibited the highest betweenness (Figure 6).

## 4. Discussion

### 4.1. No Trade-Off between Hydraulic Efficiency and Hydraulic Safety

The lack of significant correlation between P50_leaf_ and K_max_ suggests that there was no trade-off between leaf water transport efficiency and leaf embolism resistance, which differs from the results of many previous studies [10,46,47,48]. This result was consistent with the performance of woody plants in the Loess Plateau region, which is also severely affected by drought stress [49]. The absence of a trade-off relationship made it possible for plants to have both high leaf hydraulic transport efficiency and strong embolism resistance [49]. However, in this study, the leaf embolism resistance was generally weak (with high P50_leaf_ values), and severe embolism occurred during the dry season (all species showed negative HSM_leaf_ values). The generally high P50_leaf_ values led to small interspecific differences in P50_leaf_, which was one of the reasons for the lack of significant correlation between P50_leaf_ and K_max_. This also indicated that the desert shrubs in this study did not rely on strong leaf embolism resistance to maintain leaf safety. On the other hand, desert shrubs exhibited high leaf wilting resistance (TLP_leaf_ was significantly more negative than P50_leaf_) (Table 3), meaning they could maintain normal cellular morphology even in the presence of leaf embolism, which was beneficial for maintaining normal physiological activities [47,50,51]. Fang et al. (2011) found that *Caragana korshinskii* maintained leaf turgor and could still carry out photosynthetic accumulation even when exposed to extremely low water potential (below −6.1 MPa) [52]. This may be an important adaptation strategy for desert plants to arid environments. The benefit is that after leaf embolism occurs early in the drought, transpiration is reduced, thus mitigating further damage to the overall plant hydraulic system, while photosynthetic accumulation can be maintained.

The hydraulic safety (HSM_leaf_) and efficiency (K_max_) of desert shrub leaves may also be related to leaf water capacity. In this study, HSM_leaf_ was mainly related to the ability to regulate water potential (WP_leaf_) (Table S1). The ability to regulate water potential is influenced by leaf water capacity, that is, the larger the leaf water capacity, the greater the potential to buffer fluctuations in leaf water potential [53]. In this study, K_max_ was significantly positively correlated with Cm and not significantly correlated with Cs (Table S1), which is consistent with previous research results on temperate woody plants [47]. The reason may be that the leaves are not uniform “water containers”, meaning that there are hydraulic compartments within the leaves, and only a portion of the water stored in the tissues can be exchanged for transpiration [28]. The functional coordination between K_max_ and Cm explains the phenomenon that leaves with higher K_max_ close stomata more slowly when the transpiration rate is constant [54,55].

### 4.2. Leaf Economic Strategies

Desert shrubs in this study exhibited lower SLA and higher LT compared to the mean values of shrubs in other regions of China [24,25,26]. Previous studies have found that plants can improve water use efficiency by reducing SLA and increasing leaf N content in arid regions [56,57]. There may be a trade-off between water use efficiency and leaf construction costs for plants in arid regions [58]. High LT and low SLA indicate high construction costs, low growth rates, and long leaf lifespan, corresponding to the slow acquisition strategy of the plant economics spectrum [12,18]. However, in this study, there was a significant negative correlation between LT and C content, indicating that the thicker the leaf, the lower the carbon investment per unit mass of the leaf. This may be related to the leaf structure type of desert shrubs. This study found that the venation structure of thicker succulent leaves was simpler (with lower LVD), and there was a highly significant negative correlation between LT and TCf (Figure 3). In leaf tissues, leaf veins, and cuticles are constructed at a relatively high cost [59,60], contributing to improved mechanical strength and prolonged leaf life [59,61].

In conclusion, desert shrubs with thinner leaves improve leaf security by increasing resistance, while desert shrubs with thicker leaves adopt a strategy to improve water use efficiency [56,57,58,60]. Furthermore, P content was significantly positively correlated with SLA and N content in this study. In summary, species with higher SLA, N content, and P content, such as *Caragana brachypoda* and *Asterothamnus alyssoides*, adopted the strategies of fast acquisition and improved security, while species with higher LT and lower SLA and P content, such as *Zygophyllum xanthoxylum* and *Reaumuria trigyna*, adopted the strategies of slow acquisition and improved water use efficiency [62].

### 4.3. The Coupled Relationship between Leaf Hydraulic Traits and Economic Traits and Their Association with Anatomical Structure

The results of both principal component analysis and correlation analysis indicated a coupled relationship between the leaf hydraulic and economic traits of desert shrubs and that those two sets of traits were strongly correlated with anatomical structure, respectively (Figure 2 and Figure 6). Previous studies have demonstrated that such coupled correlation is a cost-effective adaptation strategy for species that contributes to resource use efficiency [26]. Higher C content and N content indicate higher leaf density and higher construction cost [63,64]. In this study, the positive correlation between C content and both WP_leaf_ and HSM_leaf_, as well as the positive correlation between the N content and HSM_tlp_, indicated that there was a synergistic relationship between leaf construction cost and hydraulic safety. This aligns with the economic theory that higher construction costs are associated with longer leaf lifespans [12]. C content was negatively correlated with Cs. A possible reason is that the space of the leaves is limited, which means that a larger water capacity requires a larger loose leaf space, resulting in a low C content value. In this study, Cs was highly significantly positively correlated with Sp (Figure 5), also indicating that a more loosely structured spongy tissue was beneficial for increasing water capacity. On the other hand, Cs was significantly negatively correlated with TCf, indicating that there was a trade-off between dense structure and water capacity. The cuticle plays a major role in preventing water loss [65]. Therefore, the negative correlation between Cs and TCf also reflected the trade-off between the leaf’s capacity for water storage and water retention. LT and Tpa were significantly negatively correlated with P50_leaf_, HSM_leaf_, and WP_leaf_, and extremely significantly positively correlated with Cs. This indicated that thicker leaves had a larger water capacity, could tolerate lower water potentials, and also required stronger leaf embolism resistance (more negative P50_leaf_) and faced a higher risk of embolism (lower HSM_leaf_). C content was extremely significantly negatively correlated with LT, Tpa, and Sp, and significantly positively correlated with TCf. WP_leaf_ and HSM_leaf_ were significantly positively correlated with TCf. This indicated that the C content in the leaf mesophyll tissue was relatively low, while the C content in the leaf cuticle layer was relatively high. Leaves with high C content and a thick cuticle layer had a safer xylem. Species with these characteristics have thinner leaves, which primarily enhance hydraulic safety by maintaining a higher water potential. Species with smaller SLA have higher water use efficiency [56,57]. Larger Sp, GCL, and SAI are more conducive to gas exchange [66,67,68]. SLA was significantly negatively correlated with Sp, GCL, and SAI, indicating that the water use efficiency and the gas exchange rate of the leaves were functionally synergistic.

In summary, LT and C content were the most strongly correlated economic traits with other leaf traits. WP_leaf_ and HSM_leaf_ were the most strongly correlated hydraulic traits with other leaf traits. Among the anatomical traits, Tpa exhibited the strongest correlation with hydraulic traits, and GCL showed the strongest correlation with economic traits (Figure 3, Figure 4 and Figure 5). In the correlation network analysis, LT had both a high degree and closeness, making it a “central trait” in the leaf trait network [69]. A previous study on angiosperms at a large scale also showed that LT was a core trait in the plant leaf trait network, playing a role in multiple functions [70]. In this study, species with thicker leaves exhibited stronger embolism resistance, larger water capacity, lower carbon investment per unit mass, and were more capable of tolerating low water potentials. Conversely, species with thinner leaves had a higher proportion of cuticles and were more capable of maintaining water potential.

## 5. Conclusions

In this study, desert shrubs exhibited weak leaf embolism resistance, low leaf hydraulic safety margins, and strong leaf wilt resistance, primarily adopting a strategy of tolerating drought stress. There was no trade-off between leaf water transport efficiency and leaf embolism resistance. On the other hand, desert shrubs were characterized by thick leaves and small specific leaf areas. Species with higher SLA, N content, and P content adopted the strategies of fast acquisition and improved security, while species with higher LT and lower SLA and P content adopted the strategies of slow acquisition and improved water use efficiency. The study emphasized the coupling relationship between the leaf hydraulic and economic traits of desert shrubs, which can be summarized as follows: (1) There was a synergistic relationship between leaf construction cost and hydraulic safety; (2) there was a trade-off between the leaf’s capacity for water storage and water retention; (3) water use efficiency was functionally synergistic with the rate of gas exchange. Furthermore, leaf anatomical structure was closely related to both leaf hydraulic and economic traits. LT was the most tightly connected with all traits and was the core trait in the leaf trait network.

In previous studies, stomatal and leaf vein anatomical traits were predominantly utilized as proxies for hydraulic traits to investigate the relationship between economic traits and hydraulic traits [24,29,31]. However, in this study, hydraulic traits such as TLP_leaf_, K_max_, and P50_leaf_ were found to be decoupled from stomatal and leaf vein anatomical traits. This finding indicates that leaf vein and stomatal traits cannot be used as a substitute for physiological leaf hydraulic traits for desert shrubs. This study investigated the relationship between typical hydraulic traits and economic traits, which can help to reveal the drought adaptation strategies of desert shrubs at a deeper level and thus provide a theoretical basis for vegetation conservation and ecological restoration in degraded desert ecosystems. Hydraulic and economic traits among different plant organs are interrelated [29]. Therefore, in future studies, we will investigate the relationship between the hydraulic and economic traits of desert shrubs from the perspective of the whole plant (including roots, stems, and leaves), with the aim of more comprehensively elaborating on the ecological adaptation strategies of desert shrubs.

## Figures and Tables

**Table 1:** Full botanical name, family, abbreviation code, leaf habit, and height of the 19 desert shrubs.

Species	Family	Code	Leaf Habit	Height (m)
*Nitraria tangutorum* Bobr	*Zygophyllaceae*	Nt	Deciduous	1–2
*Tetraena mongolica* Maxim	*Zygophyllaceae*	Tm	Deciduous	0.4–0.9
*Zygophyllum xanthoxylum* (Bunge) Maxim	*Zygophyllaceae*	Zx	Deciduous	0.5–1
*Reaumuria trigyna* Maxim	*Tamaricaceae*	Rt	Deciduous	0.1–0.3
*Prunus mongolica* (Maxim.) Ricker	*Rosaceae*	Prm	Deciduous	1–2
*Potaninia mongolica* Maxim	*Rosaceae*	Pom	Deciduous	0.3–0.4
*Atraphaxis pungens* (Bieb.) Jaub. et Spach	*Polygonaceae*	Ap	Deciduous	0.8–1.5
*Ammopiptanthus mongolicus* (Maxim. ex Kom.) Cheng f	*Fabaceae*	Am	Evergreen	1.5–2
*Caragana korshinskii* Kom	*Fabaceae*	Ck	Deciduous	1–4
*Caragana brachypoda* Pojark	*Fabaceae*	Cb	Deciduous	0.2–0.3
*Caragana tibetica* Kom	*Fabaceae*	Ct	Deciduous	0.2–0.3
*Caragana microphylla* Lam	*Fabaceae*	Cm	Deciduous	1–3
*Caragana stenophylla* Pojark	*Fabaceae*	Cs	Deciduous	0.3–0.8
*Caragana roborovskyi* Kom	*Fabaceae*	Cr	Deciduous	0.3–1
*Helianthemum songaricum* Schrenk	*Cistaceae*	Hs	Deciduous	0.1–0.12
*Krascheninnikovia ceratoides* (Linnaeus) Gueldenstaedt	*Chenopodiaceae*	Kc	Deciduous	0.1–1
*Artemisia ordosica* Krasch	*Asteraceae*	Ao	Deciduous	0.5–1
*Asterothamnus alyssoides* (Turcz.) Novopokr	*Asteraceae*	Aal	Deciduous	0.1–0.2
*Ajania achilleoides* (Turcz.) Poljakov ex Grubov	*Asteraceae*	Aac	Deciduous	0.1–0.2

**Table 2:** Basic information on 19 desert shrubs in this study.

Species	Longitude	Latitude	Association	Cover Classification	Soil Data	Soil Acid-Base Properties	Soil Sand Content (%)
*Nitraria tangutorum* Bobr	107°12′3.86″	40°13′10.84″	*Tetraena mongolica + Zygophyllum xanthoxylum*	2	Sandy brown soil	alkaline	69.5
*Tetraena mongolica* Maxim	107°12′3.86″	40°13′10.84″	*Tetraena mongolica + Zygophyllum xanthoxylum*	2	Sandy brown soil	alkaline	69.5
*Zygophyllum xanthoxylum* (Bunge) Maxim	107°12′3.86″	40°13′10.84″	*Tetraena mongolica + Zygophyllum xanthoxylum*	2	Sandy brown soil	alkaline	69.5
*Reaumuria trigyna* Maxim	107°12′3.86″	40°13′10.84″	*Tetraena mongolica + Zygophyllum xanthoxylum*	2	Sandy brown soil	alkaline	69.5
*Prunus mongolica* (Maxim.) Ricker	106°41′57.00″	40°25′43.95″	*Prunus mongolica + Ammopiptanthus mongolicus*	2	Sandy brown soil	alkaline	52.3
*Potaninia mongolica* Maxim	107°30′10.15″	40°7′28.38″	*Tetraena mongolica + Zygophyllum xanthoxylum*	2	Sandy brown soil	alkaline	69.5
*Atraphaxis pungens* (Bieb.) Jaub. et Spach	107°27′13″	39°56′27.64″	*Caragana brachypoda + Atraphaxis pungens*	2	Sandy brown soil	alkaline	75.6
*Ammopiptanthus mongolicus* (Maxim. ex Kom.) Cheng f	106°41′57.00″	40°25′43.95″	*Prunus mongolica + Ammopiptanthus mongolicus*	2	Sandy brown soil	alkaline	52.3
*Caragana korshinskii* Kom	106°41′57.00″	40°25′43.95″	*Prunus mongolica + Ammopiptanthus mongolicus*	2	Sandy brown soil	alkaline	52.3
*Caragana brachypoda* Pojark	107°27′13″	39°56′27.64″	*Caragana brachypoda + Atraphaxis pungens*	2	Sandy brown soil	alkaline	75.6
*Caragana tibetica* Kom	107°27′13″	39°56′27.64″	*Caragana brachypoda + Atraphaxis pungens*	2	Sandy brown soil	alkaline	75.6
*Caragana microphylla* Lam	107°27′13″	39°56′27.64″	*Caragana brachypoda + Atraphaxis pungens*	2	Sandy brown soil	alkaline	75.6
*Caragana stenophylla* Pojark	107°0′50.75″	39°20′29.58″	*Helianthemum songaricum + Ajania achilleoides*	2	Sandy brown soil	alkaline	51.7
*Caragana roborovskyi* Kom	107°0′50.67″	39°20′29.18″	*Helianthemum songaricum + Ajania achilleoides*	2	Sandy brown soil	alkaline	51.7
*Helianthemum songaricum* Schrenk	107°0′51.36″	39°20′29.11″	*Helianthemum songaricum + Ajania achilleoides*	2	Sandy brown soil	alkaline	51.7
*Krascheninnikovia ceratoides* (Linnaeus) Gueldenstaedt	107°27′56″	39°39′34.62″	*Artemisia ordosica + Krascheninnikovia ceratoides*	2	Sandy brown soil	alkaline	75.6
*Artemisia ordosica* Krasch	107°27′57″	39°39′34.62″	*Artemisia ordosica + Krascheninnikovia ceratoides*	3	Sandy brown soil	alkaline	75.6
*Asterothamnus alyssoides* (Turcz.) Novopokr	107°27′58″	39°39′34.62″	*Artemisia ordosica + Krascheninnikovia ceratoides*	2	Sandy brown soil	alkaline	75.6
*Ajania achilleoides* (Turcz.) Poljakov ex Grubov	107°0′50.78″	39°20′29.00″	*Helianthemum songaricum + Ajania achilleoides*	2	Gravelly brown soil	alkaline	51.7

Note: The community information was classified according to the Braun–Blanquet classification [41,42].

**Table 3:** Information on 9 leaf hydraulic traits, 6 economic traits, and 12 anatomical traits in this study.

Leaf Traits	Abbrev	Unit	Mean	Max	Min	SE
leaf vein density	LVD	mm mm^−2^	5.08	9.29	1.36	0.45
midrib diameter	MD	μm	121.71	325.03	42.40	14.87
stomatal density	SD	no. mm^−2^	285.94	525.87	121.60	8.52
average guard cell length	GCL	μm	30.22	42.53	17.90	0.49
stomatal area index	SAI	mm^2^ mm^−2^	0.25	0.42	0.08	0.01
maximum stomatal conductance	g_max_	mol m^−2^s^−1^	2.40	3.91	0.89	0.06
epidermis thickness	Tep	μm	44.81	86.72	24.12	4.05
spongy tissue thickness	Sp	μm	105.87	347.53	0.00	24.74
palisade tissue thickness	Tpa	μm	325.56	1098.17	133.32	53.84
leaf cuticle thickness	TC	μm	18.58	38.38	7.42	1.54
ratio of spongy tissue thickness to palisade tissue thickness	Sp/Tpa	%	31.72	97.70	0.00	5.96
ratio of cuticle thickness to leaf thickness	TCf	%	10.56	17.75	2.82	1.01
leaf thickness	LT	μm	482.35	1382.63	212.66	72.00
specific leaf area	SLA	m^2^ Kg^−1^	13.09	29.14	7.10	1.17
Carbon content per unit mass	C	mg g^−1^	28.47	42.39	18.05	1.53
Nitrogen content per unit mass	N	mg g^−1^	434.55	523.66	295.74	11.75
Phosphorus content per unit mass	P	mg g^−1^	1.56	2.91	0.87	0.13
the ratio of nitrogen content to phosphorus content per unit mass	N:P	—	19.39	27.71	10.36	1.11
leaf water potential at turgor loss point	TLP_leaf_	MPa	−3.81	−1.98	−6.21	0.26
maximum conductance in leaves	K_max_	mmol Kg^−1^ MPa^−1^ s^−1^	451.86	716.84	157.31	48.38
the water potential inducing 50% loss of maximum conductance in leaves	P50_leaf_	MPa	−1.01	−0.73	−1.23	0.04
leaf predawn water potential	WP_pd_	MPa	−1.36	−0.82	−2.03	0.07
leaf midday water potential	WP_leaf_	MPa	−3.16	−1.70	−4.81	0.18
leaf hydraulic safety margin	HSM_leaf_	MPa	−2.00	−0.74	−3.59	0.22
leaf hydraulic safety margins for wilting	HSM_tlp_	MPa	0.65	3.05	−1.78	0.28
leaf water capacity per unit mass	Cm	mol Kg^−1^ Pa	4.44	5.98	2.16	0.26
leaf water capacity per unit area	Cs	mol m^−2^ Pa^−1^	2.69	8.14	1.05	0.39

## Data Availability

All data and materials are available in the article and in its online Supplementary Data.

## Supplementary Materials

The following supporting information can be downloaded at: https://www.mdpi.com/article/10.3390/life14070834/s1, Table S1: Correlation between leaf hydraulic traits in desert shrubs; Table S2: Correlation between leaf economic traits in desert shrubs; Measurement methods: Measurement method for hydraulic traits. References [8,71] is cited in the supplementary materials.
