# Supplementary material for: Characteristics, Relationships, and Anatomical Basis of Leaf Hydraulic Traits and Economic Traits in Temperate Desert Shrub Species
Source: Life (Basel). 2024 Jun 29;14(7):834. doi: 10.3390/life14070834 (PMC11278145; doi:10.3390/life14070834)
Supplement: Supplementary file 1 [file life-14-00834-s001.zip › life-3067040-supplementary.pdf]

Supplementary tables

Table S1 Correlation between leaf hydraulic traits in desert shrubs

Traits	TLP_{leaf}	C_m	C_s	K_{max}	$P50_{leaf}$	WP_{pd}	WP_{leaf}	HSM_{leaf}	HSM_{tlp}
TLP_{leaf}									
C_m	-.015								
C_s	-.355	.613**							
K_{max}	-.032	.670*	-.052						
$P50_{leaf}$	.172	-.196	-.522	.381					
WP_{pd}	-.014	.330	.568*	.307	-.120				
WP_{leaf}	.220	-.007	-.462*	.123	.450	-.183			
HSM_{lesf}	.460	-.192	-.615*	.066	.308	-.158	.988**		
HSM_{tlp}	-.785**	.010	.034	.141	.218	-.104	.432	.392	

Note: * indicates significant correlation (p<0.05); ** indicates extremely significant correlation (p<0.01).

Table S2 Correlation between leaf economic traits in desert shrubs

	N	C	P	N:P	SLA	LT
N						
C	-.040					
P	.544*	.098				
N:P	.193	-.212	-.673**			
SLA	.265	.053	.766**	-.582**		
LT	-.085	-.825**	-.296	.317	-.254	

Note: * indicates significant correlation (p<0.05); ** indicates extremely significant correlation (p<0.01).

Measurement methods for hydraulic traits

(1) Leaf pressure-volume curves

Leaf turgor loss point (TLP_{leaf}, MPa) and capacitance (C, mol Kg^{-1} Pa and mol $m^{-2}Pa^{-1}$) at both pre- (C_1) and post-turgor loss (C_2) were measured following the pressure–volume method (P-V curves). Fully sun-exposed leaf-bearing branches were sampled at predawn, placed in a plastic black bag with damp towels, promptly transferred to the laboratory, and placed into a container with clean water for at least 6 hours until complete saturation. Leaves were detached from the plant with a razor blade and progressively dehydrated on a bench. During dehydration, leaf mass and water potential were measured periodically using a balance and a pressure chamber (1505D-EXP, PMS Instrument Company, Albany, OR, USA), respectively. The time intervals for the measurements were based on the rate of leaf water loss. The final measurement of leaf dry mass was conducted using an analytical balance after oven-drying at a temperature of 70 °C for a minimum of 48 hours. The P-V curve fitting program (Schulte and Hinckley, 1985) was used to calculate TLP_{leaf}.

(2) Leaf vulnerability curves

Six species (*Zygophyllum xanthoxylum*, *Atraphaxis pungens*, *Caragana stenophylla*, *Caragana roborovskyi*, *Asterothamnus alyssoides*, *Ajania achilleoides*) were excluded from leaf hydraulic vulnerability curve analysis due to their very small leaf size.

Leaf hydraulic conductance and leaf vulnerability curves were measured based on the timed rehydration method described by Brodribb and Holbrook (2003). Fully sun-exposed leaf-bearing branches were collected at predawn after rainfall events from July to September during the growing season. 5-8 healthy sun-exposed branches were collected from at least 5 individuals of each species. Upon collection, the cut ends of the branches were immediately wrapped with a wet paper towel and plastic wrap before being placed in a black plastic bag and promptly transported to the laboratory. The cut end of each branch was submerged in water, and then at least one internode length was removed to allow for rehydration over 5 hours. After rehydration, the cut end of the branch was wrapped with paraffin wax and sealed with sealing film (PM996, BEMIS, Chicago, USA). Then, the branch was placed on a dry, cool lab bench to air-dry for 10 to 30 minutes, depending on the plant's water loss rate. The branch was then left in a black plastic bag for 1-2 hours. Two mature leaves were randomly selected, and their water potential was measured. If the water potential difference between two adjacent leaves exceeded 0.2 MPa, the shoot was discarded; otherwise, the average water potential of the two adjacent leaves was considered as the initial water potential (Ψ_i, MPa). Then, two other adjacent leaves were selected, cut and rehydrated for 5 to 120 seconds based on their initial water potential. After recording the rehydration time (t, s), the leaves were placed in a self-sealing bag with wet paper towels that had no direct contact with the leaves and left in a box in the dark for 20 minutes to equilibrate before measuring the final water potential (Ψ_f, MPa). Leaf hydraulic conductance (K_{leaf}) was calculated using the following formula: $K_{leaf} = C \times \ln(\Psi_i / \Psi_f) / t$, where C represents the leaf water capacitance ($mmol \cdot m^{-2} \cdot MPa^{-1}$). If the initial water potential was less negative than the turgor loss point, C_1 was used; if the initial water potential was equal to or more negative than the turgor loss point, C_2 was used.

By repeating the above steps, a series of correlation points between Ψ_i and K_{leaf} were obtained. A sigmoidal model was then fitted using Ψ_i as the independent variable and K_{leaf} as the dependent variable. The maximum hydraulic conductance K_{max} and $P50_{leaf}$ were calculated based on the model equation.

(3) Water potential and hydraulic safety margins

Leaf midday water potential (WP_{leaf}, MPa) and predawn water potential (WP_{pd}, MPa) measurements were conducted using a pressure chamber (1505D-EXP, PMS Instrument Company, Albany, OR, USA). 10 fully sun-exposed leaf-bearing branches and healthy leaves from 5 individuals per species were measured before dawn ((WP_{pd}, MPa, 5:30–6:30) and at mid-afternoon and mid-afternoon (WP_{leaf}, MPa, 12:00–14:00) on continuously sunn

y days on September 2022.

Leaf hydraulic safety margins for wilting (HSM_{tlp}, MPa) were calculated as the difference between Ψ_{leaf} and TLP_{leaf}. Leaf hydraulic safety margins for embolism (HSM_{leaf}, MPa) were calculated as the difference between Ψ_{leaf} and $P50_{leaf}$.

References:

Brodribb TJ, Holbrook NM (2003) Stomatal closure during leaf dehydration, correlation with other leaf physiological traits. Plant Physiol 132(4): 2166–2173.

Schulte PJ, Hinckley TM (1985) A comparison of pressure-volume curve data analysis techniques. J Exp Bot 36(10): 590–602.
